# Application of spectroscopic methods for direct characterization of photosynthetic pigments and inert intracellular components in the model purple non sulfur bacterium *Rhodospirillum rubrum*

**DOI:** 10.1186/s12934-025-02876-w

**Published:** 2025-12-14

**Authors:** Eva Slaninova, Viktorie-Alexandra Pacasova, Ota Samek, Hugo Fleuriot-Blitman, Manfred Zinn, Martin Koller, Marketa Benesova, Stanislav Obruca, Petr Sedlacek

**Affiliations:** 1 Faculty of Chemistry, Brno University of Technology, Purkynova 464/118, 612 00 Brno, Czech Republic; 2 Institute of Scientific Instruments, v.v.i., Czech Academy of Sciences, Kralovopolska 147, 612 64 Brno, Czech Republic; 3 Institute of Life Sciences, University of Applied Sciences and Arts Western Switzerland Valais-Wallis (HES-SO Valais-Wallis), Sion, Switzerland; 4 Institute of Chemistry, NAWI Graz, University of Graz, Heinrichstrasse 28/IV, 8010 Graz, Austria

**Keywords:** *Rhodospirillum rubrum*, Photosynthetic pigments, Carotenoids, Bacteriochlorophyll *a*, Polyhydroxyalkanoates, UV-Vis spectroscopy, Vibrational spectroscopy

## Abstract

**Background:**

Non-invasive spectroscopic methods are increasingly valued in life sciences, where preserving the native state of biomolecules is essential for accurate interpretation. Traditional analyses of microbial compounds typically involve solvent-based extraction and chromatographic separation processes, which are time consuming, damaging to samples, and can alter biomolecular structures of complexes. To overcome these limitations, we developed a novel spectroscopic workflow for direct metabolite monitoring in microbial cells.

**Results:**

In this study, we established a combined spectroscopic methodology that allows direct pigment and polyhydroxyalkanoates (PHAs) analysis in complex biological samples without requiring chemical extraction procedures. The UV-Vis spectroscopy technique using an integrating sphere enables direct monitoring of pigments even in turbid whole cell suspensions, providing detailed fingerprints of bacteriochlorophyll *a* and carotenoids in their natural environment. Together, these techniques provide consistent information about cellular composition. Using the photosynthetic bacterium *Rhodospirillum rubrum* as a model organism, we demonstrate that our combined spectroscopic approach can resolve pigment states, reveal intracellular PHA content and crystallinity, and measure carotenoids and bacteriochlorophylls directly in native whole cell suspensions. Furthermore, advanced data processing provided an improved interpretation of pigment and PHA states in different cellular forms.

**Conclusions:**

This innovative combination of spectroscopic techniques reduces sample manipulation, preserves cellular integrity and provides rapid, precise, and environmentally friendly analysis of microbial metabolites in their natural physiological conditions. The demonstrated workflow is broadly applicable to biological samples where maintaining biomolecular integrity is crucial, and it has strong potential for applications in process analytical technology and industrial biotechnology.

**Graphical Abstract:**

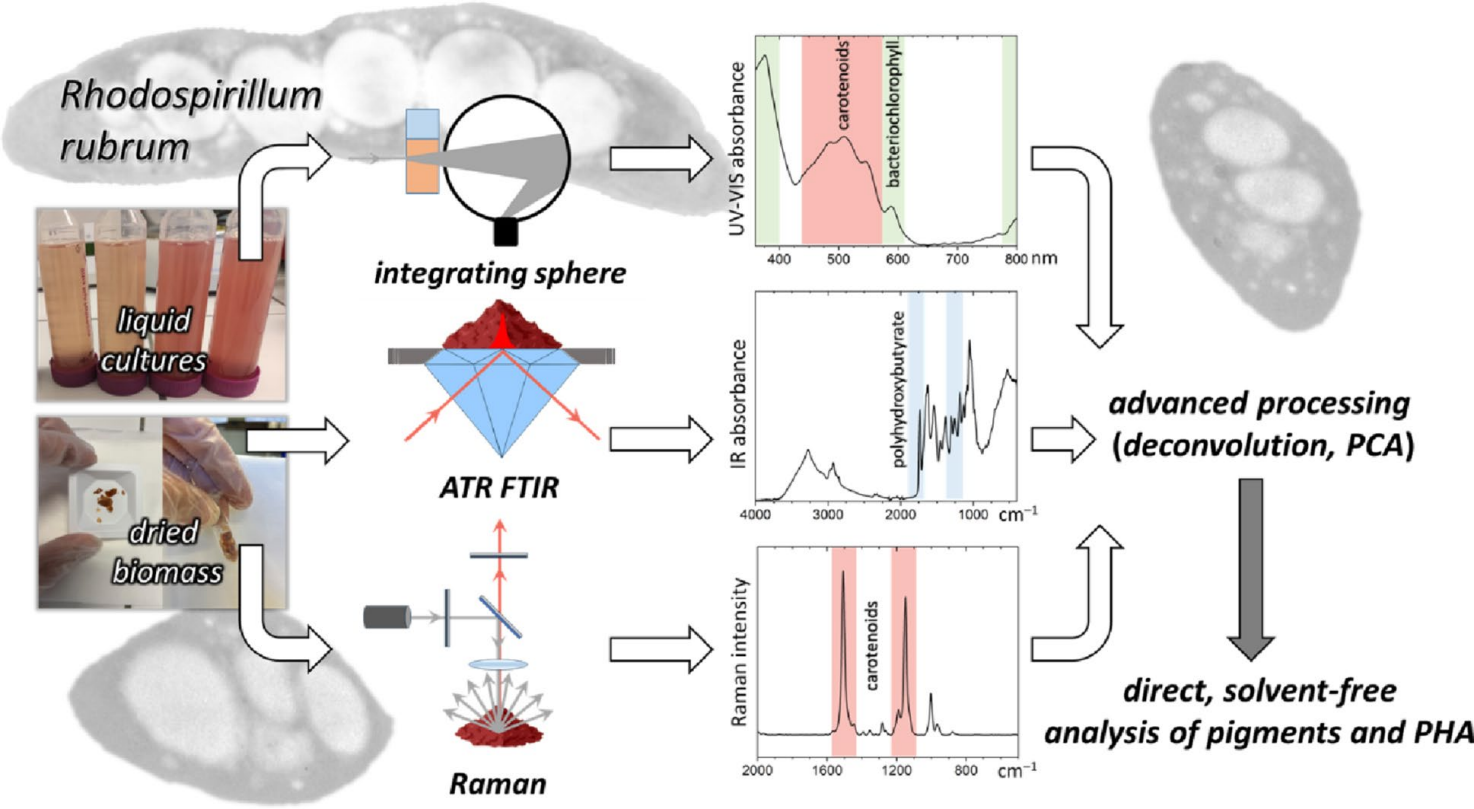

## Background

Photosynthetic microorganisms are essential components of Earth’s biosphere, playing a foundational role in global biogeochemical cycles. Among them, photosynthetic bacteria, especially purple non sulfur bacteria (PNSB) such as *Rhodospirillum rubrum*, contribute significantly to carbon fixation, nitrogen cycling, and energy flow in both aquatic and terrestrial ecosystems. These organisms perform anoxygenic photosynthesis, utilizing light energy to drive electron transport without producing oxygen, often employing organic acids or molecular hydrogen as electron donors. Their metabolic activity is especially important in stratified environments such as sediments, microbial mats, and anaerobic zones of freshwater bodies, where they support nutrient regeneration and energy transformation [1, 2]. *R. rubrum* is widely used as a model organism in photosynthesis research due to its genetic tractability and well-characterized photosynthetic machinery, and as role model to study regulatory pathways of nitrogen fixation systems [3, 4]. Its use in fundamental studies continues to advance our understanding of microbial metabolism, bioenergetics, and phototrophic processes.

In addition to its ecological significance, *R. rubrum*, which has the ability to fix nitrogen due to nitrogenase activity, has also emerged as a valuable organism for biotechnological exploitation. Owing to its exceptional metabolic versatility, it can grow under both aerobic and anaerobic conditions, with or without light, and utilize a wide variety of organic and inorganic carbon sources. This adaptability enables the production of valuable bioproducts such as polyhydroxyalkanoates (PHAs), molecular hydrogen, carotenoid pigments, coenzyme Q10, and 5-aminolevulinic acid, which are compounds with applications in the pharmaceutical, agricultural, and cosmetic sectors, and, regarding molecular hydrogen, also as renewable energy carrier [3, 5, 6].

Among these bioproducts, carotenoid pigments are a notable output of the photosynthetic apparatus. *R. rubrum* synthesizes bacteriochlorophyll *a* (BChl *a*) and carotenoids of the spheroidene class such as spheroidene, spheroidenone, and spirilloxanthin, which play essential roles in light harvesting and photoprotection. These pigments also hold market potential as natural colorants and antioxidants. For example, Wang et al. [7] demonstrated that *R. rubrum* could accumulate lycopene, a pigment also known from the plant kingdom as the characteristic colorant of tomatoes, at high levels of up to 2 mg per gram of dry cell weight or 15 mgs per liter of culture under dark, microaerobic, high cell density conditions, which are levels comparable to those of engineered *Escherichia coli* strains.

Another important group of bioproducts is PHAs, a family of biodegradable polyesters that are accumulated predominately by prokaryotes from various genera intracellularly under conditions of carbon surplus and nutrient limitation [8]. PHAs are biocompatible and biodegradable polymers, which make them attractive for use in various fields such as sustainable packaging, agricultural films, and biomedical applications [9, 10]. Their biosynthesis by photosynthetic bacteria is especially advantageous because it can be driven by light energy and does not compete with carbon sources relevant to human nutrition. In addition to serving as carbon and energy storage compounds, PHAs also play a protective role by helping cells resist environmental stress factors such as ultraviolet radiation, osmotic imbalance, or extreme temperatures [11].

To fully exploit the potential of *R. rubrum* in both fundamental research and industrial production, it is necessary to accurately monitor the biosynthesis of key metabolites such as pigments and PHAs. Conventional analytical methods rely on solvent-based extraction of pigments followed by UV-Vis spectrophotometry, while PHA quantification typically involves gas chromatography or high-performance liquid chromatography. Both techniques require laborious sample pretreatment: GC analysis involves acidic alcoholysis and transesterification of PHAs after drying microbial biomass [12], whereas HPLC methods to determine PHAs are based on prior alkaline digestion of the culture broth [13]. Although these techniques are well established, they are inherently destructive, labor- and material-intensive and time demanding. Moreover, they are susceptible to analytical variability resulting from sample degradation, product loss during handling, or incomplete extraction [14–16].

In this study, we present a combined spectroscopic strategy for monitoring the intracellular production of both carotenoid pigments and PHAs in *R. rubrum.* We employed UV-Vis spectroscopy with a sphere integrating scattered light to analyze both solvent-extracted pigments and intact turbid cultures enabling direct comparison between conventional and non-invasive measurements. This setup allowed us for *at line* assessment of pigment accumulation without disrupting cellular structure or requiring complex sample preparation or extraction. In parallel, we applied Attenuated Total Reflectance Fourier-Transform Infrared (ATR-FTIR) and Raman spectroscopy on dried biomass to gain complementary molecular level insight into pigment and PHA composition. By combining these three spectroscopic methods, we established a flexible, robust and reliable workflow enabling rapid screening of the content of the selected metabolites in cultures of *R. rubrum* with detailed chemical/structure characterization.

## Materials and methods

### Microorganisms and cultivation

A lyophilized bacterial culture of *Rhodospirillum rubrum* DSM 467 was obtained from the Leibnitz Institute DSMZ-German Collection of Microorganism and Cell Cultures, Braunschweig, Germany. Cultivation was performed in three steps. In the first step, the strain was incubated on LB agar plates (tryptone 10.0 g/L, yeast extract 5.0 g/L, NaCl 5.0 g/L) at 30 °C in the dark for 5 d. In the second step, 500 mL Erlenmeyer flasks containing 100 mL of LB medium were inoculated with single-colony biomass collected from the agar plates and incubated at 30 °C in the dark under continuous shaking (160 rpm) until an optical density (OD_660_) of 1.0 was achieved. The third step involved inoculation of the main culture to OD_660_ = 0.02 in modified RRNCO (*R. rubrum* no-light carbon monoxide) medium [17]. The composition of 1 L medium was as follows: 250 mg of MgSO_4_·7H_2_O, 132 mg of CaCl_2_·2H_2_O, 1 g of NH_4_Cl, 2.1 g of MOPS buffer, 1.0 g of yeast extract, 20 µM NiSO_4_, 10 mL of a chelated iron-molybdenum solution (prepared from 0.28 g H_3_BO_3_, 2 g of Na_2_EDTA, 0.4 g of FeSO_4_, and 0.1 g of Na_2_MoO_4_ per liter of distilled water), 2 µg of biotin, 1 mL of a sterile-filtered phosphate buffer solution (K₂HPO₄/KH₂PO₄ at 95.5 mM, pH 7), 10 mL of 1.5 M fructose solution (15 mM in total) and 10 mL of 1 M sodium acetate solution (10 mM in total). The pH-value of the medium was adjusted to 7.1 using NaOH. The cultures prepared in this way were cultivated in 50 mL tubes containing 45 mL of the modified RRNCO medium at 30 °C. For pigment production, cultures were incubated under daylight conditions for 72, 120, and 168 h. For PHA production, cultures were incubated in the darkness for 48, 72, and 96 h in 500 mL Erlenmeyer flasks containing 100 mL of medium with the same medium composition, except for the carbon sources (fructose and acetate) which was replaced by 30 mL of 1 M acetate solution to achieve a final concentration of 300 mM acetate in 100 mL medium, and in 500 mL Erlenmeyer flasks.

### Analysis of PHA

To determine PHA content in biomass, 20 mL samples were centrifuged (rcf: 3773 g/5 min), and the resulting biomass was washed with PBS buffer (8 g/L of NaCl, 0.2 g/L of KCl, 1.44 g/L of Na_2_KHPO_4_, 0.24 g/L of KH_2_PO_4_) adjusted to pH 7.5 with NaOH. The samples were centrifuged again and dried overnight in a thermostat at 70 °C. The PHA content in cells was then analyzed as methyl esters of 3-hydroxyalkanoates by gas chromatography, as described previously [18].

### Standard extraction-based quantification of microbial pigments

For analysing the content of bacteriochlorophyll *a* (BChl *a*) and carotenoids via methanol extraction, an established protocol for pigment extraction from cyanobacteria was used [19]. Absorbances of the extracts were measured spectrophotometrically (Nanophotometer Implen P300, Germany) at a wavelength of 470 nm, 665 nm, and 720 nm. To calculate pigment concentrations of BChl *a* and carotenoids, we used corresponding Eqs. (1–2) [20, 21]:

1 $$ {\mathrm{BChl}}\;a\left[ {{\upmu }{\mathrm{g}}/{\mathrm{mL}}} \right] = 12.9447 \left( {A_{{665}} - A_{{720}} } \right) $$

2 $$ \begin{aligned} & {\mathrm{Carotenoids}}\;\left[ {{\upmu }{\mathrm{g}}/{\mathrm{mL}}} \right] = \\ & \quad \left[ {1000\left( {A_{{470}} - A_{{720}} } \right) - 2.86\left( {{\mathrm{BChl}}\;a\left[ {{\upmu }{\mathrm{g}}/{\mathrm{mL}}} \right]} \right)} \right]/221 \\ \end{aligned} $$

### Direct UV-Vis spectroscopy of cell suspensions

UV-Vis spectroscopy characterization of the undiluted suspensions of cells and their extracts were performed by UV-Vis absorption spectrophotometry (U-3900 H, Hitachi, Japan) both in a regular turbidimetry mode and also in diffuse reflection mode (light-integrating sphere attachment 60mmDIA for Hitachi U-3900 H spectrophotometer) at scan speed of 600 nm/min from wavelength of 300 to 900 nm [22].

### Spectroscopic analyses of dried microbial biomass

#### Fourier transform infrared spectroscopy

For further structural analysis of the dried biomass, Fourier-transform infrared (FTIR) spectroscopy was employed. FTIR spectra of biomass dried to constant weight at 70 °C were recorded using an iS50 FTIR spectrometer (Thermo Scientific, Waltham, MA, USA). All measurements were conducted at ambient temperature using the built-in single-reflection diamond attenuated total reflectance (ATR) accessory. Each absorption spectrum was obtained as the average of 32 scans at a resolution of 4 cm^–1^ with data spacing of 0.5 cm^–1^. For each cultured biomass type, spectra were collected from at least seven independent samples. Additional spectra were obtained when increased sample heterogeneity was observed, particularly for biomass collected after 72 h under light and 48 h under dark cultivation conditions. The mean spectrum was calculated as the arithmetic average of these individual spectra. Principal component analysis (PCA) of all measured spectra was performed using a standard multivariate algorithm developed in-house with MATLAB software (MathWorks, Natick, MA, USA) at the Institute of Scientific Instruments, Czech Academy of Sciences [23]. Prior to analysis, all spectra were background-corrected using the rolling circle method (circle radius: 1000 cm^–1^; 10 passes) and normalized to the height of the Amide II peak (1531 cm^–1^). To minimize artifacts originating from the diamond ATR crystal and residual water content, the spectral regions 850–1800 cm^–1^ and 2800–4000 cm^–1^ were selected for PCA.

#### Raman spectroscopy

Aside from FTIR, Raman spectroscopy was used to determine the fingerprint of pigments in whole cells. For this technique, the washed suspensions of cells were pipetted on CaF_2_ (Crystran, UK) in three parallel spots. After drying at room temperature, the samples were measured by Renishaw Invia system (Renishaw Invia Raman spectrometer, UK), using a 532 nm single-mode diode laser as the Raman excitation light. The laser beam was focused by microscope objective (50×, NA 0.5 Leica, Germany) where the spot has a diameter of approximately 2 × 10 μm, each spot was measured 10 times with acquisition time of 10s. Obtained spectra were acquired in the range of 500–2000 cm^−1^ [24]. FT-Raman spectra were recorded using FT-Raman module with iS50 FTIR spectrometer (Thermo Scientific). The module is equipped with a 1064 nm excitation laser with software-controlled power settings. Each spectrum was recorded as the average of 1024 scans at a resolution of 8 cm⁻¹. An excitation laser power of 0.1 mW was used for all measurements to achieve an optimal balance between signal intensity and background fluorescence. Prior to further analysis, the Raman spectra were background-corrected using the Rolling Circle algorithm (radius: 250; number of passes: 5–10).

## Results and discussion

### Characteristics of the cultivation processes

Spectroscopic techniques offer numerous advantages in microbial biotechnology, such as high robustness, the ability to detect and quantify multiple metabolites, and generally fast and reliable operation, all of which are crucial criteria [25]. To evaluate the selected spectroscopic methods, we prepared cultures of *R. rubrum* under carefully controlled conditions. The goal was to obtain samples as similar as possible in most characteristics except for their pigment content, specifically carotenoids and BChl *a*, and their PHA content. We cultivated the bacterial strain *R. rubrum* under two different conditions: continuous illumination and complete darkness (see Materials and Methods). These conditions are known to have a strong effect on pigment production [26, 27]. To determine the actual pigment concentrations in the samples (shown in Table 1), we analyzed selected pigments using a standard solvent extraction method (see Materials and Methods).

It is well known that *R. rubrum* produces higher amounts of PHA when grown in the dark with excess carbon sources such as volatile fatty acids (VFAs), organic acids or sugars (in case of fructose, production also depends on dissolved oxygen level) [28, 29]. Although the main focus of this study was pigment detection, we also included PHAs as an additional point of interest because the spectroscopic methods can detect both pigments and intracellular PHAs directly in bacterial biomass. To avoid distortion in UV-Vis spectra by PHA granules, which could affect the quality of the spectroscopic data, we intentionally limited PHA production during light cultivation. Nevertheless, PHA were not excluded from our analyses completely (shown in Table 1). Since their synthesis is triggered under specific conditions such as darkness and the presence of suitable carbon sources, we also monitored them in selected dark-grown cultures [17, 28, 30]. For comparing the spectra of individual samples with varying PHA content, the actual PHA concentrations were firstly determined by gas chromatography.

Based on our controlled cultivation conditions, we obtained samples with high amounts of pigments, as well as increased amounts of PHA even at low cell density (see in Table 1). The pigmented samples were cultivated under microaerobic conditions that support anoxygenic photosynthesis due to the activation of chromatophores, which caused that the bacterial culture exhibited a distinct purplish-red color. In contrast, under dark aerobic conditions, the presence of oxygen induced inhibition of pigment synthesis in the photosynthetic apparatus and the cells appeared colorless [31]. However, we set up our experiment to achieve these different samples not only through environmental conditions, but also by selecting carbon substrates. Specifically, acetate was chosen to induce higher PHA accumulation, as it is known to result in slower bacterial growth, during which metabolic shift occurs from biomass production towards PHA storage [32, 33]. Nevertheless, even the samples cultivated microaerobically under illumination did not reach a high OD. In this case, the low cell density might be due to the initially high light intensity applied immediately after inoculation, which might have resulted in light inhibition at an early stage of cultivation.

**Table 1:** Basic growth and composition characteristics of the cultures used for the spectroscopic analyses

	Time (h)	OD_660_	PHA (% CDW)	c_BChl a_ (µg/mL)	c_carotenoids_ (µg/mL)
Daylight cultivation	72	0.43 ± 0.01	0.89	0.02 ± 0.01	0.66 ± 0.01
	120	0.93 ± 0.01	0.21	0.05 ± 0.00	1.74 ± 0.33
	168	0.86 ± 0.10	0.08	0.11 ± 0.08	3.46 ± 0.26
Dark cultivation	48	0.18 ± 0.01	14.33	n.d.	n.d.
	72	0.91 ± 0.13	25.21	n.d.	n.d.
	96	0.25 ± 0.01	9.48	n.d.	n.d.

*n.d.* not detected, *CDW* cell dry weight

### Characterization of the cell suspensions by spectrophotometry

**Fig. 1:**
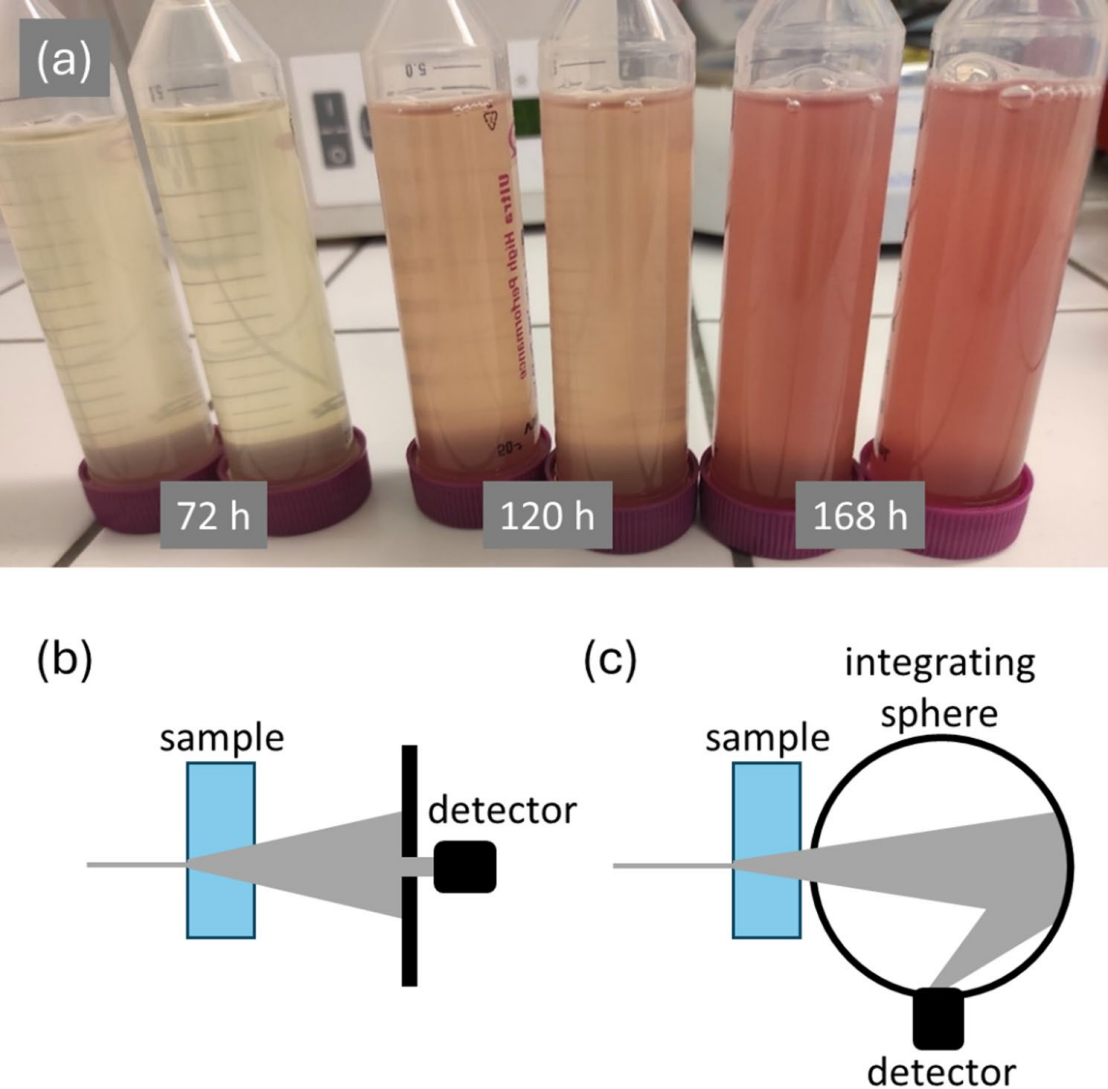
In situ spectrophotometric characterization of *R. rubrum* in cell dispersions. **a** Fresh bacterial cultures of *R. rubrum* cultivated under light conditions. **b** Schematic representation of standard transmission spectrophotometry. **c** Schematic representation of the integrating sphere spectrophotometry

**Fig. 2:**
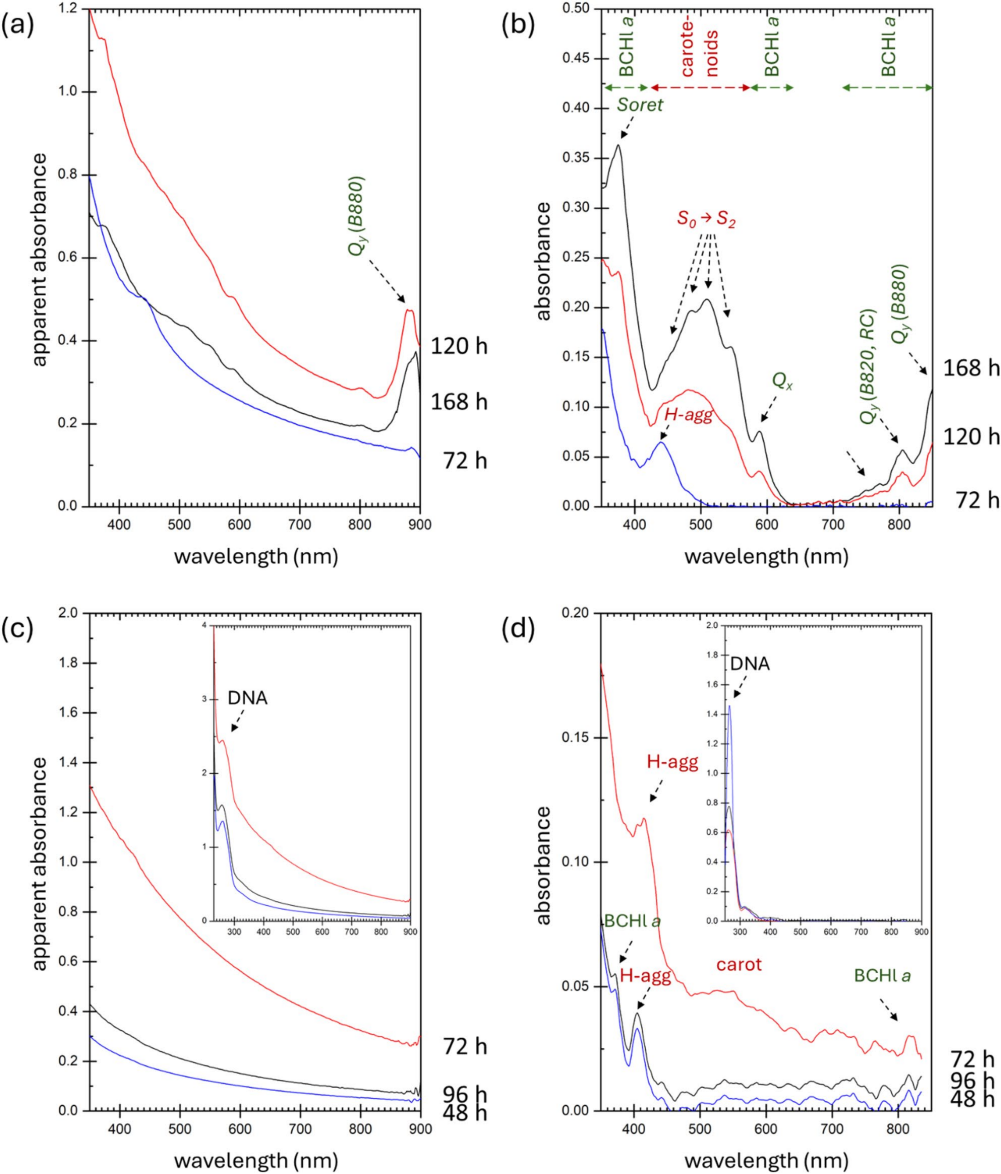
Results of direct UV-Vis spectrophotometric analysis of *R. rubrum* cultivated under illumination for different times. **a**, **b** and under dark conditions **c**, **d**. The spectra were obtained by: **a**, **c** standard transmission spectrophotometry, **b**, **d** integrating sphere spectrophotometry

Two spectrophotometry techniques were used for the characterization of cell suspensions of *R. rubrum* (a schematic representation of the techniques is shown in Fig. 1). Results of the spectrophotometric analyses of the cell cultures, cultivated either in the light or in the dark condition, are shown in Fig. 2. As expected, UV-Vis spectra recorded by the standard transmission spectrophotometry are dominated by the scattering of light by the bacterial cells (see Fig. 2a, c). The scattering signal increases in parallel with the number of scattering particles. Actually, this technique is therefore routinely used for monitoring the growth of microbial cultures. Moreover, because the light scatters not only on the cell surface, we have demonstrated in our previous work that the standard transition spectrophotometry can be employed also for studying the light scattering caused by the cell ultrastructure (e.g., by submicron-sized intracellular inclusion such as PHA granules) [22]. Moreover, the strong background scattering signal limits direct spectrophotometric monitoring of specific absorption of other cellular components including photosynthetic pigments. As can be seen in spectra shown in Fig. 2a, among all the specific absorption bands of bacterial photosynthetic pigments, only the near-infrared absorption at about 880 nm is clearly visible in the transmission spectra of photosynthesizing bacteria. For this reason, we have involved the technique of integrating sphere spectrophotometry (alternatively called Diffuse-transmission spectroscopy) in our study. This technique employs a spherical mirror that spatially integrates the scattered light (Fig. 1c) and hence allows for correct measurement of light absorption even in highly opaque samples. Although the light-integrating sphere accessories are readily available commercially and the technique has a long tradition of use in analyses of liquid colloids [34], its utilization in analysis of microbial research is still relatively scarce. Only recently, the technique was proposed as a valuable tool also for a solvent-free pigment analysis in algae [35, 36] and cyanobacteria [37].

The low pigment content in the cultures cultivated in the dark revealed by the extraction procedure was confirmed also by the *at line* spectrophotometry as represented by the poor absorption signal in the visible region (Fig. 2d). On the other hand, for the *R. rubrum* culture cultivated under continuous illumination, it is obvious that the integrating sphere spectroscopy provides good resolution absorption spectra, thus giving a detailed information on the electronic properties of the photosynthetic apparatus of the bacterium (Fig. 2b) directly in vivo. The culture cultivated for the longest time (168 h) shows a complex absorption spectrum with the detailed spectral fingerprint of the whole supramolecular photosynthetic assembly of the cell that combines the light-harvesting complex (LH1) and reaction center (RC). The detailed structure of the LH1-RC complex of *R. rubrum* has recently been described with the use of Cryo-EM [38]. Absorption spectra of organic extracts of LH1-RC complex as well as its constituents were published as well [39, 40]. The *at line* spectrum obtained here corresponds well with these published results. Obviously, the spectrum translates by the electronic transitions that can be easily assigned to both pigment components of the photosynthetic complex—bacteriochlorophyll a (BChl *a*), and a member of the carotenoid family, spirilloxanthin. Both pigments are involved as an integral part of the light-harvesting complex LH1. In *R. rubrum* cells, this complex forms a closed, slightly elliptical double ring that surrounds the reaction center (RC). The double ring contains 16 pairs of polypeptides (inner α, outer β), whereby one molecule of all-trans-spirilloxanthin and two BChl *a* molecules belong to a single polypeptide pair [38]. An additional pair of BChl *a* is also ligated in the RC, while no carotenoids are located there. A basic assignment of the individual absorption bands of the integrating sphere spectrum is indicated in Fig. 2b.

Presence of BChl *a* provides the spectrum with typical electronic transition of bacteriochlorins mainly in the blue-to-UV and red-to-NIR regions [41]. The major singlet electronic transitions that occur on the porphyrin skeleton are referred to as Soret (alternatively called B-type), Qx and Qy transitions. Q-type absorption bands stand for the first two π → π* transitions that are mutually different in the direction of the polarization of the excited porphyrin ring, while the composite Soret band is composed of several electron transitions to higher excited states. Similarly, spirilloxanthin contained in LH1 brings in the spectrum recorded for the longest time of cultivation (168 h) the typical absorption fingerprint of carotenoids, located in the blue and green (400–600 nm) region. The complex carotenoid absorption band represents by the electronic transitions of the π-conjugated polyene motifs from the ground to the second excited state (S_0_ → S_2_), as far as the first excited state is absorption silent [42]. In this situation, the characteristic three-peak structure of the carotenoid absorption band corresponds to the transitions to the lowest three vibronic states of S_2_. Importantly, the actual position and shape of all above discussed absorption peaks is strongly affected not only by the chemical composition of the pigments, but also by the molecular configuration and physico-chemical properties (e.g., polarity, polarizability) of their surroundings. Hence, the *at line*/direct spectrophotometric analysis of the bacterial pigments can provide valuable information not only on pigments metabolism per se (identification of the pigment molecules synthesized in the cells) but also on the pigment localization in the cell and on the way how they are bound in the architecture of specific cellular components.

This can be illustrated in further analysis of the absorption signals of both types of pigments in spectra shown in Fig. 2b. In the case of BChl *a*, the Qy band can serve as a good indicator of the type of complexation of the pigment molecule. It is well known that while the monomeric form of BChl *a* absorbs at about 777 nm, exciton pair transition of BChl *a* dimer, ligation of the BCHL *a* dimer with polypeptides, as well as its hydrophobic, π–π and polar conjugation with carotenoids, lead to a progressive red shift of this band in the cellular environment. Hence, in the whole-cell spectra shown in Fig. 2, the shoulder at 777 nm (marked with arrow in Fig. 2b) represents the small population of unbound BChl *a* molecules [39], the band at 820 nm is explained by the single BChl *a* pair conjugated in the RC [40] and for the LH1 structural subunit complex (depicted as B820) [38–40], while the most red-shifted band at 880 nm corresponds to the BChl *a* electronic transitions in the fully assembled LH1 system [38]. Similarly, the carotenoids reveal the typical vibronic states-resolved spectrum only from the non-polar local environments such as those provided by the lipidic membranes or by conjugation with hydrophobic domains of polypeptides. Therefore, the three-band pattern of S_0_ → S_2_ electronic transition in spectra represent carotenoid pigments located in the fully assembled LH1 complex. In addition, in a polar medium, carotenoid molecules are known to self-aggregate, forming either the strongly “card-like” packed (H-type), or the weakly coupled (J-type) aggregates, whereby the type of the aggregation depends primarily on the molecular structure of the pigment [43]. The H-type aggregation results in exactly those changes in the absorption spectrum (strong blue-shift and the loss of the vibronic state resolution) [44] that were observed in the spectrum of *R. rubrum* cultivated under light conditions for 72 h. Hence, this spectrophotometric observation indicates that in the initial stages of the *R. rubrum* cultivation, carotenoid pigments are distributed in the aqueous cell environment mainly in the non-ligated form, providing the cell with other functions beyond the energy-harvesting (e.g., photo-protective and/or antioxidant), thus facilitating the adaptation of the culture to the cultivation conditions. Interestingly, it was found that also for the cultures cultivated in the dark, the residual content of carotenoids (not detectable by the extraction techniques) shows specific spectral features of the H-aggregates dispersed in aqueous media. This again supports the assumption that under circumstances where photosynthesis does not represent the principal metabolic strategy, the carotenoid-like pigments are synthesized in the cell to serve in another than a simple energy-harvesting way. Furthermore, it also provides a clear picture of the benefits of the extraction-free detection of pigments in the cell using *at line* analysis.

Beyond offering a qualitative overview of pigment content and the identification of pigment components in cell cultures, integrating sphere spectrophotometry also provides a robust foundation for deeper quantitative analysis. This technique has been demonstrated as a powerful, solvent-free method for accurately quantifying total pigment content in whole cells [36]. Moreover, it facilitates the differentiation of various microorganisms in mixed cultures. For example, in unicellular photosynthetic microorganisms containing chlorophylls and bacteriochlorophylls, this method successfully distinguished pigments such as BChl *c* and BChl *a*, which are otherwise poorly resolved using solvent-based spectroscopy [45].

**Fig. 3:**
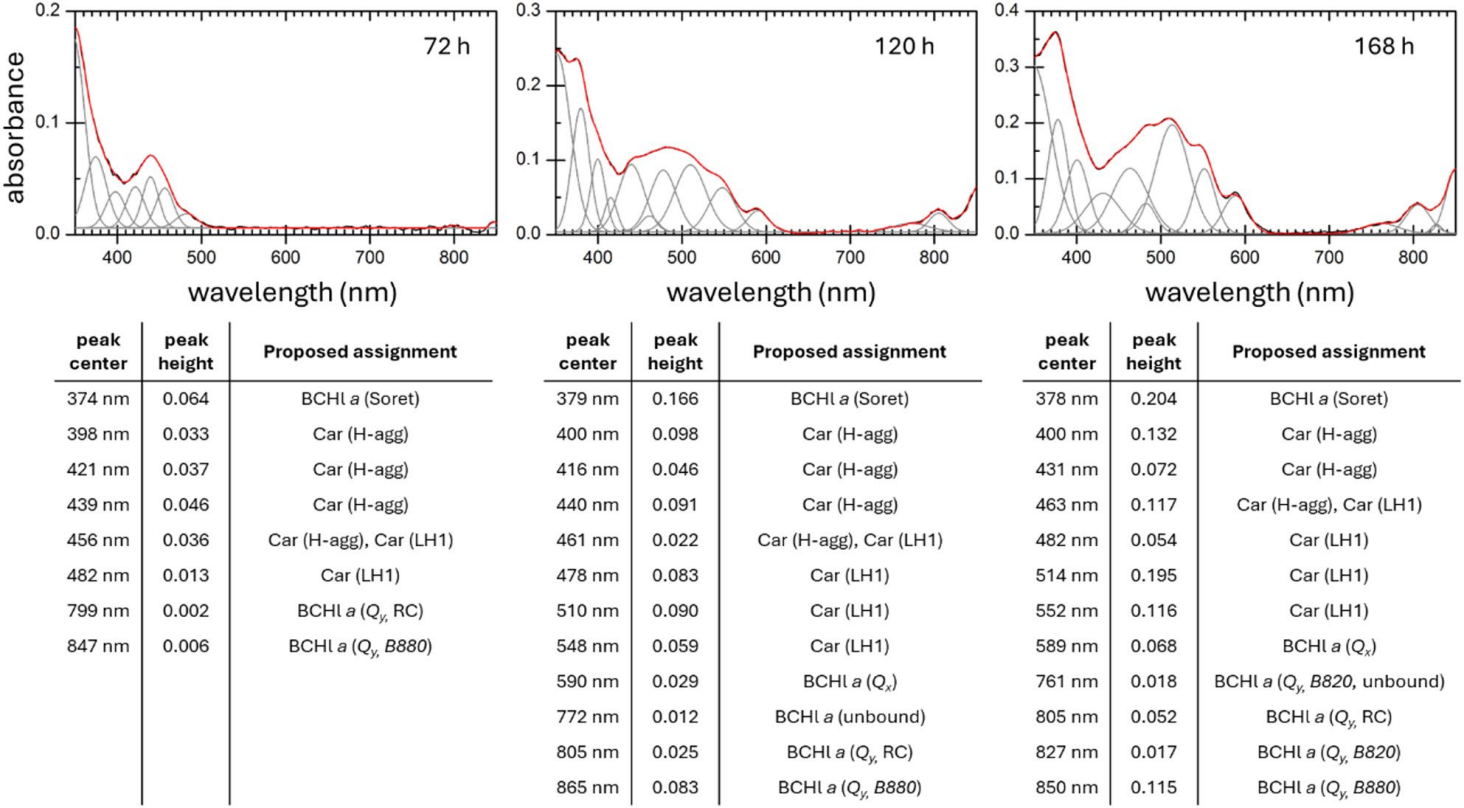
Spectral deconvolution of in vivo UV–Vis absorption spectra of *R. rubrum* cultivated under illumination. Deconvoluted in vivo UV–Vis spectra of *R. rubrum* cells grown under illumination for three cultivation times, measured using an integrating-sphere setup. The identified components correspond to distinct pigment states: monomeric BChl *a* (777 nm), RC bound dimers (820 nm) and LH1 integrated BChl *a* (880 nm). In the carotenoid region (400–600 nm), the vibrational structure in the samples indicates spirilloxanthin integration into LH1, whereas its absence or broadening in early stages suggests H-type aggregation of free spirilloxanthin

In systems with more complex pigment compositions, such as our *R. rubrum* cultures, the quantitative capabilities of this method can be further enhanced through advanced data processing techniques, particularly spectral deconvolution. This approach, which involves decomposing the overall UV-Vis spectrum into individual absorption components, significantly improves the resolution and identification of overlapping spectral features, revealing hidden transitions and pigment species. Furthermore, deconvolution enables the use of advanced mathematical modeling and chemometric techniques, including partial least squares regression and multivariate curve resolution [46], as well as optimization algorithms such as Bayesian LARS-OLS (Least Angle Regression-Ordinary Least Square) [47]. When combined with machine learning, these strategies support real-time contaminant monitoring and automation of cultivation processes, thereby improving the stability and viability of phototrophic cultures [48]. An example of deconvolution applied to the integrating sphere spectra of the *R. rubrum* cultures is shown in Fig. 3. This analysis provides both qualitative and basic quantitative insight, with the peak heights representing the relative contributions of individual pigment constituents and assemblies identified within the overall UV-Vis spectrum.

### Analysis of dried biomass using infrared spectroscopy

**Fig. 4:**
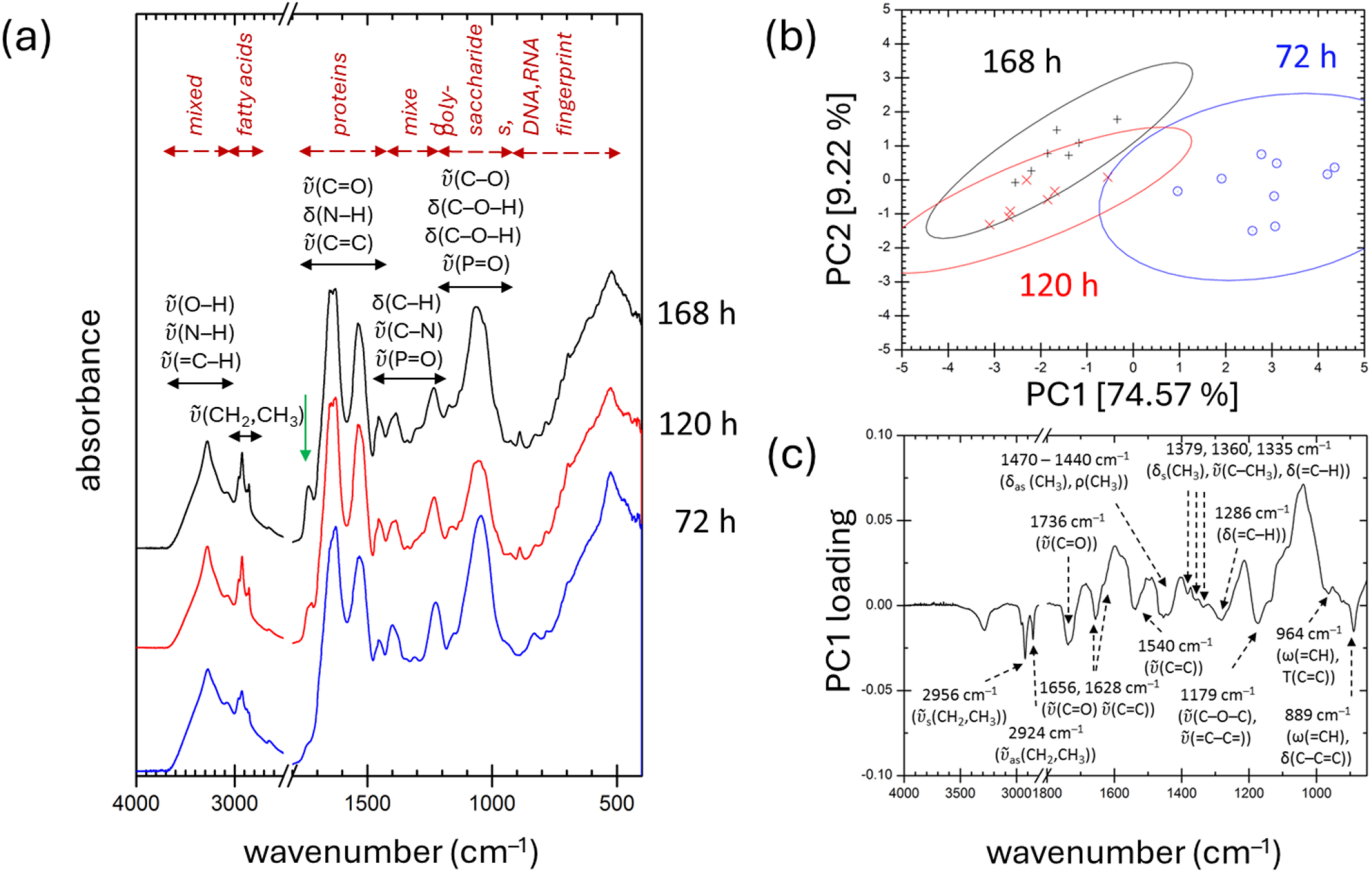
ATR-FTIR analysis of dried *R. rubrum* biomass cultivated under light conditions. **a** Mean ATR-FTIR spectra recorded at different time points (72, 120, and 168 h). Major spectral features are annotated, and the carbonyl (C=O) stretching band is highlighted with a green arrow. **b** Principal Component Analysis (PCA) score biplot showing sample clustering based on spectral similarities. The values in square brackets indicate the percentage of variance explained by each principal component. Ellipses represent 95% confidence intervals. **c** Loading plot of PC1, illustrating the spectral features contributing most to the separation of samples along this component

**Fig. 5:**
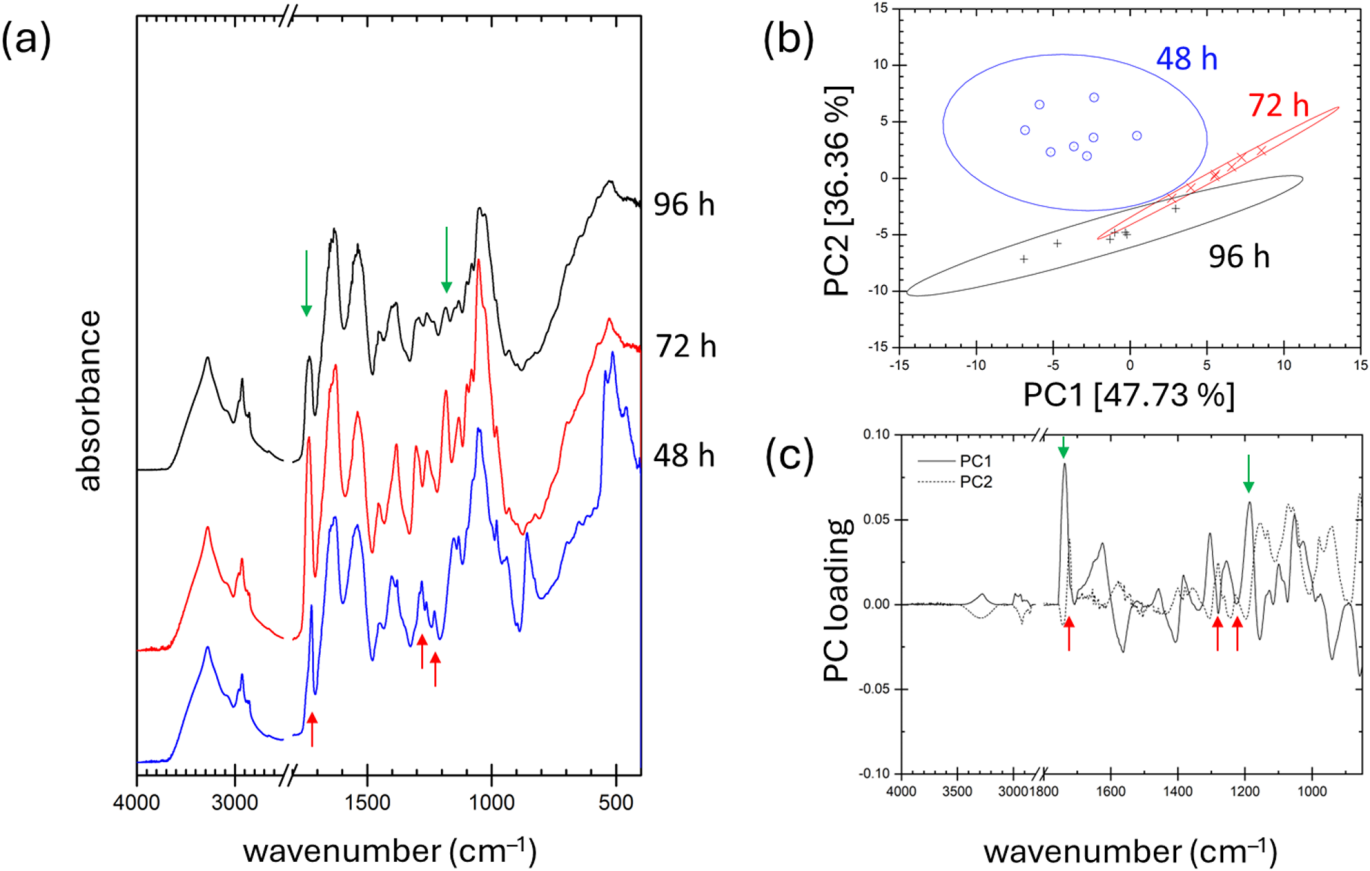
ATR-FTIR analysis of dried *R. rubrum* biomass cultivated under dark conditions. **a** Mean ATR-FTIR spectra recorded at different time points (48, 72, and 96 h). PHA-specific bands are highlighted with arrows. **b** Principal Component Analysis (PCA) score biplot showing sample clustering based on spectral similarities. The values in square brackets indicate the percentage of variance explained by each principal component. Ellipses represent 95% confidence intervals. **c** Loading plot of PC1 and PC2, illustrating the spectral features contributing most to the separation of samples along this component

FTIR and Raman spectroscopy are well-established, non-destructive analytical techniques widely used for the characterization of biological materials, including pigment extracts [49, 50]. In recent years, their application has expanded to specific advanced analyses, particularly for compounds such as carotenoids and PHAs [51–53]. In the present study, we employed ATR-FTIR spectroscopy to analyze dried *R. rubrum* biomass samples. For each culture condition, spectra were recorded from at least seven independent biomass subsamples. The resulting spectra, representing cultures grown under light and dark conditions at various time points, are presented in Figs. 4 and 5. These spectra exhibit characteristic features corresponding to the major structural components of bacterial biomass (see assignments in Fig. 5) [54].

At first glance, only minor differences are apparent in the spectra shown in Fig. 4. These differences likely reflect variations in the content and composition of photosynthetic complexes across the samples. In particular, the intensities of the carbonyl (C=O) stretching band at ~ 1740 cm^−1^ (highlighted by the green arrow in Fig. 4) and the aliphatic C–H stretching region just below 3000 cm^−1^ appear to correlate with the content of extracted pigments (see Table 1). While the strong C–H stretching pattern is characteristic of bacterial carotenoids [55], the origin of the carbonyl band is less straightforward. Although this vibration is a typical feature of PHAs [56], its assignment to PHAs is not supported by the GC analysis, which confirmed their absence. A carbonyl signal associated with pigments has been reported in some cyclopentanone-containing compounds [57]; however, we consider it more likely that the observed band originates from intracytoplasmic lipids present in the membranes of pigment-containing chromatophores [58, 59]. Thus, although FTIR spectra capture some pigment-related signals, they do not provide conclusive in situ evidence for the presence of photosynthetic pigments in the biomass.

Nonetheless, more subtle structural differences between light-grown samples collected at different cultivation times become evident when the FTIR spectra are subjected to Principal Component Analysis (PCA). This underexplored statistical method allows for the detection of underlying patterns and spectral variation among samples. The PCA results are shown in Fig. 4b and c. As illustrated in Fig. 4b, the PCA score biplot reveals clear clustering: Samples from the shortest cultivation period (72 h) group separates along the positive axis of Principal Component 1 (PC1), while spectra from the 120- and 168-h time points largely overlap in the negative PC1 region. The corresponding PC1 loading plot (Fig. 4c) highlights the spectral features driving this separation. Notably, these variations correspond to vibrations attributed to carotenoids, bacteriochlorophylls, and associated protein components of the photosynthetic complex (see Fig. 4 for vibrational assignments, based on literature [57, 60]).

Figure 5 presents the results of ATR-FTIR analysis of *R. rubrum* cultures grown under dark conditions. In contrast to the light-grown cultures, the spectra of dark-cultivated samples collected at different time points show more pronounced differences, particularly in the intensity of vibrational bands associated with PHAs. Notably, the PHA content in intact cells is reflected in the increased absorbance of the carbonyl (C=O) stretching band around 1740 cm^−1^ and the C–O–C stretching band near 1180 cm^−1^—both highlighted by green arrows in Fig. 5.

Beyond providing semi-quantitative information on overall PHA accumulation, our previous work has shown that FTIR signatures of PHAs are also highly sensitive to changes in polymer crystallinity. This makes it possible to monitor the extent of cell lysis and stress induced crystallization of native intracellular PHAs [53]. Specifically, deformation of the carbonyl band—manifested as peak sharpening and a shift toward lower frequencies—along with the appearance of new absorption bands characteristic of PHA crystallites (at ~ 1280 and ~ 1230 cm^−1^), indicates a higher proportion of lysed cells in the 48-h culture compared to the older samples. These crystallinity-sensitive spectral features are highlighted with red arrows in Fig. 5.

Once again, the value of applying advanced multivariate analysis is evident. PCA of the FTIR data not only distinguishes the cultures based on overall PHA content (as captured by Principal Component 1), but also reflects differences in PHA crystallinity (captured by Principal Component 2), as shown in Fig. 5b and c.

### In situ analysis with Raman spectroscopy

As mentioned in the previous section, Raman spectroscopy is a non-destructive method and can be effectively used for the analysis of bacterial biomass including the detection of pigments and PHAs. According to previous studies, this method was already used as a stand-alone technique for bacterial biomass analysis [24, 61]. Compared to FTIR, Raman spectroscopy offers several advantages, such as lower sensitivity to water, the ability to use optical probes and the possibility of direct measurements in liquid samples.

**Fig. 6:**
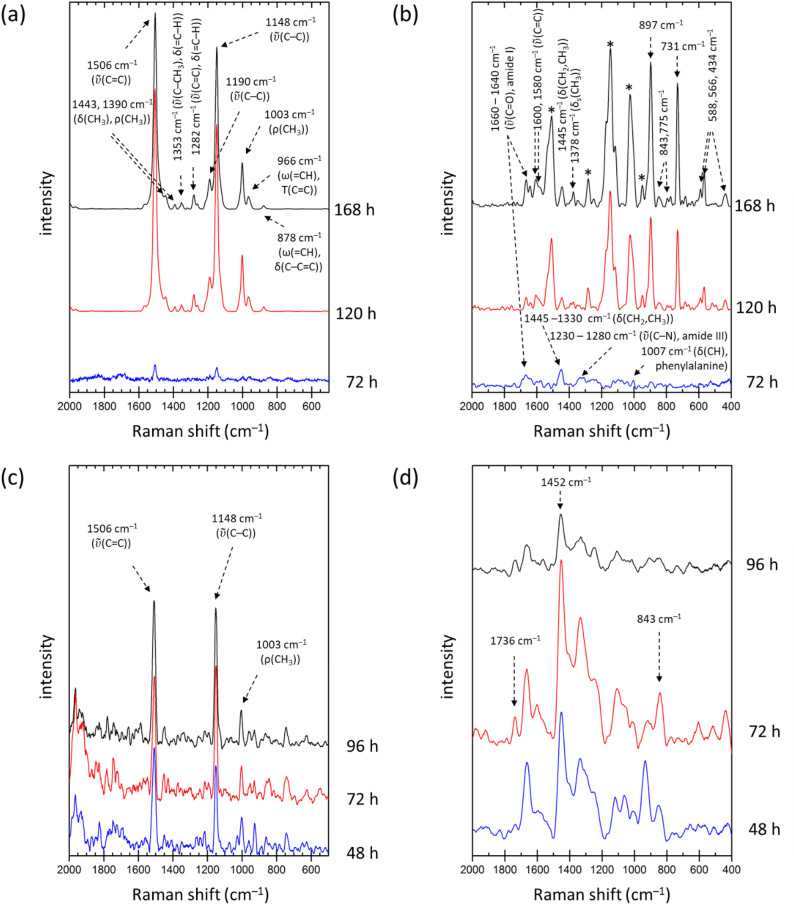
Raman analysis of dried *R. rubrum* biomass. Dispersive Raman microspectroscopy (**a**, **c**) and FT-Raman spectroscopy (**b**, **d**) of samples cultivated under light (**a**, **b**) and dark (**c**, **d**) conditions

In this study, we employed two variants of Raman spectroscopy to analyze *R. rubrum* biomass, dispersive Raman microspectroscopy and FT-Raman spectrometry, and compared their ability to detect specific metabolic compounds. The resulting spectra are shown in Fig. 6. Distinct differences are evident not only between cultures grown under different conditions but also between both Raman techniques used.

The primary distinction between these techniques lies in the excitation wavelength. This difference is particularly important when analyzing colored samples, where the excitation wavelength overlaps with an electronic absorption band of the analyte. This principle underlies Resonance Raman spectroscopy, which enhances the sensitivity of conventional Raman methods. Resonance selectively amplifies vibrational signals associated with chromophores, enabling detection of low-abundance species, offering structural insights into specific molecular subunits, and improving spectral contrast in complex biological or chemical matrices.

In our experiments, the dispersive Raman microscope uses a green laser (532 nm), which resonates with the electronic transitions of carotenoids. In contrast, the FT-Raman spectrometer employs near-infrared excitation (1064 nm), which interacts pre-resonantly with bacteriochlorophylls. This difference is clearly reflected in Fig. 6a and b. Under green excitation, the Raman spectra of *R. rubrum* are dominated by signals from carotenoids. These spectral features are assigned based on the previous works on the topic [55, 62, 63]. As expected, the intensity of the Raman signal increases with carotenoid content in the cultures.

Conversely, in FT-Raman spectra of the same samples, the carotenoid-specific bands (marked with asterisks) are significantly reduced, revealing underlying spectral features from other cellular components. Notably, distinct Raman bands associated with bacteriochlorophylls are observed, such as ring-stretching modes around 1600 cm^−1^, out-of-plane bending modes between 700 and 1000 cm^−1^, and metal-ligand stretching and ring-puckering vibrations below 600 cm^−1^ [64].

For cultures grown in the dark, Raman spectra acquired with green excitation are of low quality due to the reduced carotenoid content. In contrast, FT-Raman spectra from these samples display distinct and sufficiently intense signals of PHAs, characterized by bands at 1736, 1452, and 843 cm^−1^. These features align with our previous work on Raman-based identification of PHAs in bacterial cells [24].

### Cross-evaluation of the methods

Unlike previous studies that typically focus on a single compound, metabolite or in situ/in vivo analytical method, several recent investigations have addressed broader groups of related biomolecules within the same pigment or biopolymer family, such as carotenoids [50], chlorophylls including bacteriochlorophyll [65], or even PHAs in mixed cultures [66]. In contrast, we propose combination of spectroscopic techniques to detect and monitor different cellular components within one experimental setup.

Direct spectroscopic techniques offer a non-destructive alternative for analyzing intact biomass, thereby streamlining workflows, reducing use of chemicals, and preserving the biological context of the sample. Methods such as UV-Vis, Fourier-transform infrared (FTIR), and Raman spectroscopy have shown considerable promise for real-time monitoring of intracellular pigments and PHAs [67, 68]. In industrial applications, these approaches are increasingly implemented as part of Process Analytical Technology (PAT) approaches, allowing real-time control and optimization. Raman spectroscopy, for example, provides *in line* and non-invasive molecular-level monitoring with high selectivity and minimal interference from water [69]. FT-Raman spectroscopy has also been successfully applied to track carotenoid accumulation and substrate consumption in microbial fermentation systems [70]. Moreover, FTIR spectroscopy provided advanced interpretation of complex spectra, however overlapping spectral bands often present challenge and require further analysis such as PCA or PLS (Partial Least Squares regression) [51]. These findings were also confirmed by our results, where ATR-FTIR and Raman spectroscopy were successfully employed to detect key intracellular components directly in the samples, even with different metabolite content such as pigments (BChl *a*, carotenoids) and PHAs. Specifically, PHAs were clearly detected in the FTIR spectra and intracellular pigments were detected by Raman with a different laser. In addition, by applying PCA analysis, we were able to obtain advanced interpretation of spectral differences among individual samples. Although specific PAT-based studies on *R. rubrum* pigments are still limited, insights from related microbial systems suggest that the integration of in situ Raman or near-infrared (NIR) probes could significantly improve process control, yield optimization, and scalability.

While advanced spectroscopic platforms provide high-resolution chemical information, they often require significant financial investment and complex integration procedures. For smaller-scale research or early process development, cost-effective alternatives can be highly valuable. One such option is the use of integrating spheres in combination with benchtop UV-Vis spectrophotometers. Compared to fiber-optic or Raman probes, integrating spheres are easier to implement, require less maintenance, and are considerably less expensive. They are especially suitable for rapid screening, routine culture monitoring, and method development in resource-limited settings [71, 72]. In addition, the integration also provides a significant advantage in the analysis of optically dense or turbid samples, such as submersed microbial cultures where conventional transmission-based methods often show experimental limitations [22]. Based on our results, we proved that the implementation of integrating spheres is very useful for spectral measurements of turbid bacterial suspensions, where pigment signals remain unaffected by solvent extractions. Moreover, due to spectral deconvolution, we resolved individual absorption signals corresponding to specific chemical bonds, which are strongly influenced by their environment. When UV-Vis spectroscopy techniques are applied directly to living cultures without compromising cellular integrity, they may also be referred to as “*at line* methods” because they allow observation of dynamic biological processes in their natural state/structure. This combined *at line* and in vivo capability is particularly useful in microbial biotechnology, where non-invasive and fast monitoring supports both fundamental research and applied process optimization. The ability to analyze such complex samples containing pigments and PHAs directly, without prior extraction or separation, represents unique advantage and shows the value of these spectroscopic techniques for non-destructive applications.

## Conclusions

This study demonstrates that combination of UV-Vis, ATR-FTIR and Raman spectroscopy enables direct and reproducible monitoring of pigments and PHAs in *R. rubrum*, which represents a complex biological system driven by a versatile metabolic apparatus. Using an integrating sphere, we obtained in vivo absorption fingerprints of bacteriochlorophyll *a* and carotenoids in their native state, even in turbid suspensions. Vibrational spectroscopy of dried biomass provided additional molecular insights, including the detection of PHA content and crystallinity. The method also revealed structural details of the light-harvesting complex, including specific spectral signatures related to assembled pigment-protein complexes. This methodology can be applied to microbial cultures without requiring solvent extraction, making it suitable for routine monitoring and process control. In a broader perspective, our strategy could be used in industrial biotechnology and process technology to enable real-time analysis using an integrating sphere. This combination of spectroscopic techniques therefore opens new opportunities for studying complex biological samples and biotechnological applications.

## Data Availability

Data will be made available on request from the corresponding author.
